# Recent Advances in Anticancer Strategies

**DOI:** 10.3390/cancers17020173

**Published:** 2025-01-08

**Authors:** Zhiwei Hu, Hassan Bousbaa

**Affiliations:** 1 Pelotonia Institute for Immuno-Oncology, The Arthur G. James Comprehensive Cancer Center, The Ohio State University College of Medicine, Columbus, OH 43210, USA; 2 UNIPRO—Oral Pathology and Rehabilitation Research Unit, University Institute of Health Sciences (IUCS), Cooperativa de Ensino Superior Politécnico e Universitário (CESPU), Rua Central de Gandra, 1317, 4585-116 Gandra, Portugal

Due to the intricate nature of cancer development and progression, various types of cancer are increasingly prevalent worldwide. Despite radiotherapy and chemotherapy remaining the primary treatment options, their conventional approaches are plagued by significant toxicity and resistance issues, resulting in incomplete tumor eradication [1]. This presents a distinct challenge for researchers and clinicians alike. Therefore, there is an urgent need for new anticancer drugs and innovative drug delivery strategies to address these shortcomings and potentially offer more effective and safer therapeutic alternatives. This first edition of the topic “Recent Advances in Anticancer Strategies” (https://www.mdpi.com/topics/A8U6WXLXT6; accessed on 16 December 2024), focuses on pioneering research into the development and validation of novel anticancer approaches that could have a significant clinical impact in the near future.

Thirty papers were published, comprising thirteen articles and seventeen reviews, showcasing the latest advancements in novel anticancer approaches. This Editorial provides a concise summary of the findings and key highlights from these publications.

Antibody–drug conjugates (ADCs) represent a transformative approach in cancer therapy, utilizing antibodies to deliver cytotoxic drugs directly to cancer cells [2]. Research has highlighted advancements in ADCs for the treatment of gynecological cancers, dual inhibition strategies for oral cancer, and innovative drug delivery systems using functionalized calcium carbonate-based microparticles. These developments aim to enhance therapeutic outcomes by reducing off-target effects and improving the specificity and efficiency of drug delivery (Contributions 1–3).

The field of targeted therapies continues to advance with the development of kinase inhibitors for cancer bioimaging and therapy, as well as strategies targeting EGFR and glucose metabolism [3]. These approaches demonstrate potential in overcoming drug resistance and improving treatment outcomes. The synergistic targeting of EGFR and spindle assembly checkpoint pathways in oral cancer, alongside the utilization of fluorescent kinase inhibitors, are key examples of how precision medicine can be integrated into cancer care (Contributions 4–7).

Genetic and epigenetic research is uncovering biomarkers that could significantly impact cancer treatment [4]. Studies have identified key genes associated with prostate cancer progression, HER2-negative breast cancer responses, and cytarabine resistance in acute myeloid leukemia. These findings pave the way for more personalized therapies based on individual genetic profiles, optimizing treatment regimens and enhancing survival rates (Contributions 8–10). Slika et al. provide a comprehensive overview of the molecular and genetic underpinnings of medulloblastoma, detailing the distinct neurodevelopmental pathways and genetic mutations associated with different subgroups of the disease. They highlight how these molecular features can be leveraged to identify new therapeutic targets and inform treatment strategies (Contribution 11). Suba examines the role of DNA damage responses in tumors, emphasizing their impact on cellular processes beyond mere proliferation and highlighting the necessity for supportive medical care (Contribution 12).

Cancer immunotherapy is increasingly focusing on the development of bispecific antibodies, immune cell engagers, chimeric antigen receptor (CAR)-modified T and NK cells (CAR-T and CAR-NK), overcoming resistance mechanisms and harnessing the immune system’s full potential [5,6]. Strategies include the use of immune checkpoint inhibitors, microbial contributions to neoantigen immunity, and restoring apoptosis in cancers such as colorectal cancer. These approaches represent a shift towards leveraging the body’s immune defenses to fight cancer, offering new hope for patients with otherwise resistant forms of the disease (Contributions 13, 14).

Innovations in pharmacology and drug delivery are crucial for improving cancer treatment outcomes [7]. The research highlights the potential of gold nanoparticles for targeted pancreatic cancer therapy, as well as the benefits of laparoscopic versus robotic-assisted surgery for colon cancer. New formulations for LED-based photo-chemotherapy are also showing promise, providing more effective drug delivery and treatment options for skin cancer (Contributions 15–17). Su et al. explore the synergistic therapeutic potential of curcumin and baicalin co-loaded nanoliposomes for the treatment of non-small cell lung cancer, emphasizing the importance of formulation strategies for enhancing drug efficacy and overcoming resistance (Contribution 18).

Understanding the underlying mechanisms of cancer cell biology is essential for the development of new therapeutic strategies [8]. Recent studies have explored the cytotoxic effects of doxorubicin on cancer cells and the role of mitochondrial dynamics in non-apoptotic cell death, and targeted cancer stem cells in colorectal cancer. These insights are crucial for developing therapies that address the complexity of cancer cell biology (Contributions 19–21). FLASH radiotherapy involves the high-dose-rate delivery of radiation in very short pulses, which minimizes damage to surrounding healthy tissues while potentially increasing the radiosensitivity of cancer cells. The review article by Siddique et al. discusses the use of advanced radiation dosimeters to measure these high-dose rates accurately, which is critical for optimizing therapy and understanding the biological effects of FLASH radiotherapy (Contribution 22).

Optimizing treatment outcomes through clinical trials and novel therapies remains a priority [9]. The research includes the validation of predictive biomarkers used in cancer clinical trials, the management of neuroendocrine neoplasms of unknown primary origin, and the use of combination therapies involving local ablative techniques with radiotherapy. These studies aim to refine therapeutic strategies, enhance efficacy, and improve patient quality of life (Contributions 23–25). Koning et al. reviewed various intraoperative techniques used to accurately define the mucosal margins of oral cancer, thereby assisting surgeons in achieving complete resection (Contribution 26). Morin et al. present a single-institution retrospective study comparing weekly paclitaxel regimens in recurrent platinum-resistant ovarian cancer. Their analysis offers valuable insights into the efficacy and outcomes of different treatment schedules, which are crucial for optimizing therapeutic strategies and improving patient prognosis in gynecological cancers (Contribution 27). Volpe et al. discuss the latest advances in managing radioactive iodine-refractory differentiated thyroid cancer, focusing on new therapeutic strategies and their implications for patient outcomes. This research is crucial for optimizing treatment protocols and improving prognosis for patients with this challenging cancer type (Contribution 28).

Clinical trials and real-world evidence are vital for validating new cancer therapies and improving patient care [10]. Advances in radioligand therapy for metastatic castration-resistant prostate cancer, comparisons of laparoscopic versus robotic-assisted surgery for colon cancer, and an understanding of the prognostic factors in metastatic urothelial carcinoma are all critical areas of focus. These studies contribute valuable data, informing clinical practice and guiding treatment decisions (Contributions 16, 29, and 30).

This inaugural edition of the topic “Recent Advances in Anticancer Strategies” highlights groundbreaking research and diverse perspectives within the field of cancer treatment. The integration of novel therapies, targeted interventions, and innovative approaches discussed in these papers reflects the dynamic landscape of oncology. The success of this first edition not only demonstrates the depth of the scientific inquiry and collaboration in this area but also motivates us to continue advancing this discourse. We are pleased to announce that the call for submissions for the second edition is already open (https://www.mdpi.com/topics/1MJ7OBGQ2R; accessed on 18 December 2024); researchers are invited to contribute their latest findings and ideas, including but not limited to the discovery of novel tumor targets and cancer cell pathways, new bi/multi-specific antibodies, immune cell engagers, ADCs, tumor-specific CAR or T cell receptor (TCR)-modified immune cells, tumor-targeting photodynamic and radiation diagnoses and therapies, new approaches to eliminating immune suppressor cells (such as regulatory T cells, Treg; tumor-associated macrophages, TAM; myeloid-derived suppressor cells, MDSC; cancer-associated fibroblast, and CAF), cancer stem cells, and tumor neovasculature, to further shape the future of cancer research and clinical practice.
